# The Impact of COVID-19 on Self-Reported Substance Use, Well-Being, and Functioning Among United States Veterans: A Cross-Sectional Study

**DOI:** 10.3389/fpsyg.2022.812247

**Published:** 2022-04-11

**Authors:** Erin D. Reilly, Elizabeth S. Chamberlin, Brooke A. Duarte, J. Irene Harris, Steven D. Shirk, Megan M. Kelly

**Affiliations:** ^1^Mental Illness Research, Education, and Clinical Center (MIRECC), VA Bedford Healthcare System, Bedford, MA, United States; ^2^The Department of Psychiatry and Department of Population and Quantitative Health Sciences, University of Massachusetts Medical School, Worcester, MA, United States; ^3^Suffolk University, Boston, MA, United States; ^4^Department of Psychiatry, University of Minnesota Medical School, Minneapolis, MN, United States

**Keywords:** substance use disorders, addiction, veterans, pandemic (COVID19), functioning

## Abstract

As the COVID-19 pandemic sweeps the globe, many veterans with substance use issues have faced the closure of treatment facilities, mandates to shelter in place, and social distancing measures. To better understand their pandemic experiences, substance use changes, and functioning, a survey was nationally administered to a sample of United States veterans reporting substance use issues during the pandemic. The purpose of this cross-sectional online survey for veterans (*N* = 409) was to report on COVID-19 experiences, safety behaviors, and infection experiences while also investigating the relationship among addictive behaviors, mental and physical health, and COVID-19 impact. Measures also assessed specific substance use concerns, pandemic-related loneliness, and functioning. Though few veterans reported personally receiving a confirmed COVID-19 medical diagnosis (10.5%), the impact of pandemic stressors was evident, with a majority reporting anxiety related to contracting COVID-19 (61.4%) or fear of a family member or close friend contracting COVID-19 (58.7%). Participants reported increased use of alcohol (45.3%), sedatives (36.6%), inhalants (35.7%), tobacco (35.0%), and cannabis (34.9%), attributed specifically to the pandemic. Regression analyses revealed that even when controlling for the contribution of problematic substance use issues, negative pandemic impacts and self-reported COVID-19 related loneliness were related to more impaired physical and mental health functioning during the pandemic. Findings from this sample of veterans with addiction issues add to the growing literature suggesting unique and adverse effects of COVID-19 stressors on functioning while also revealing specific pandemic impacts for this group.

## Introduction

Since March 2020, the SARS-CoV-2 (COVID-19) pandemic has been an intense stressor across the United States. In the first year of the pandemic, sudden and dramatic behavioral changes were either mandated or encouraged to “flatten the curve,” which included asking people to stay at home as much as possible, shutting down entire industries (e.g., restaurants and movie theaters), and shifting clinical resources to COVID-19 specific care (Emerick et al., 2020). While these shifts were intended to slow infection rates, there was also a negative peripheral impact for many citizens due to sudden job loss (Matthews et al., 2021), reduced social connectedness (Talcott et al., 2021), and loss of certain health treatments (Hochstatter et al., 2021). Across the globe, there were also reports of growing psychological distress such as heightened suicidal ideation (Brailovskaia et al., 2021), increased negative affective states such as nervousness and fear (Zhang et al., 2020), an increased sense of worry and loneliness (Cohn-Schwartz et al., 2021), and decreased coping resources (Mana et al., 2021). Pandemic stressors and negative psychological impact may have led to societal challenges exceeding individual coping resources, likely also contributing to reports of allostatic overload (Cosci and Guidi, 2021), or the cumulative impact of an individual’s accumulated burden of stress on the body and mind (Danese and McEwen, 2012). Since allostatic overload can have downstream negative health consequences, it is unsurprising that at the beginning of the pandemic many citizens, with and without reported COVID-19 infections, reported both decreased mental health (Czeisler et al., 2020) and physical functioning (Morlock et al., 2021).

At the same time, the first few months of the pandemic saw increases in alcohol sales (The Nielsen Company, 2020) and self-reported use of some drugs (Janulis et al., 2021). In accordance with the motivational model of substance use (Cooper et al., 1995), there was concern that heightened pandemic-related psychological distress, combined with a lack of internal and external resources for stress and mood management, was leading to increased substance misuse by individuals seeking both positive affective states and coping with negative emotional experiences (Cooper et al., 1995, 2016). As COVID-19 stressors have the potential to increase substance misuse and health issues in vulnerable populations (Du et al., 2020), the Veterans Health Administration (VHA) became concerned with the impact of COVID-19 stressors on veterans’ drinking and drug use (Insider, 2020). Substance use is a significant problem among United States military veterans (Oliva et al., 2017; Teeters et al., 2017) and is associated with numerous harmful effects, including adverse physical and mental health functioning (Boden and Hoggatt, 2018). Recent COVID-19 research also supports this trend, as veterans’ increases in substance use have been associated with corresponding decreases in mental and physical health (Roberts et al., 2021).

Factors predictive of veteran functioning include more than merely problematic substance use. An important framework to consider when assessing the functional impact of substance misuse for veterans is the unique social conditions of their lives, referred to by the World Health Organization (WHO) as social determinants of health (SDH; World Health Organization, 2021). Social determinants include both structural determinants (e.g., income, finances, and age), and intermediary determinants (e.g., psychosocial circumstances and social support; Hosseini Shokouh et al., 2017), which can strongly influence health outcomes. The pandemic has had well-recorded and massive impacts on SDH areas, including changes in employment patterns, social networks, and reported quality of life (Guerin et al., 2021; Niles et al., 2021; Rogers et al., 2021). Conditions reflecting disadvantaged SDH contexts have been widely associated with psychosocial stressors and mental health issues (Allen et al., 2014). These SDH and environmental conditions can result in allostatic load issues and worse health outcomes (Thisted, 2003; Denny and Brownell, 2010). Attempts to reconcile the unbalanced number of stressors without adequate access to societal and personal coping resources may have an interactive effect with negative coping strategies such as problematic substance use, further negatively impacting the functioning of veterans.

The combined impact of these factors—pandemic impacts on quality of life (e.g., finances and meeting basic needs)—on the health and well-being of veterans is not yet clear. Though there have been studies on the impact of COVID-19 on veteran substance use (Fitzke et al., 2021; Pedersen et al., 2021), none have specifically investigated problematic substance use, COVID-19 related changes in SDH, and functional outcomes. There is also a continued and growing need to understand more precisely COVID-19 health, stressors, and infection-control behaviors, which have occurred in very particular contexts for individuals as they balance maintaining their quality of life and staying safe. For example, social distancing can increase safety from COVID-19 infection; however, loneliness is a risk factor for relapse (Volkow, 2020) because it triggers irritability, anxiety, fear, sadness, anger, or boredom (Ornell et al., 2020). These specific COVID-19 experiences for veterans are essential to consider when investigating the impact of COVID-19, substance use concerns, and health outcomes.

### The Current Study

The main aim of the present study was to investigate the impact of the COVID-19 pandemic on United States veterans who self-reported problematic drug and alcohol use. Within the framework of SDH, the study specifically assessed veterans’ lived experience of COVID-19 management, SDH (COVID-19 related quality of life indicators and loneliness), and their mental and physical health functioning. This included a particular aim to examine the contribution of SDH factors in the relationship between problematic substance use and functioning. The planned stepwise hierarchical regression model originally included *a priori* Hypotheses 1 and 2 (i.e., hypothesized prior to data collection but not publicly pre-registered). An additional *post hoc* Hypothesis 3 was added following data collection to examine a potential interaction between negative COVID-19 impacts on quality of life (e.g., finances and meeting basic needs) and COVID-19-related loneliness.

### Hypotheses

*Hypothesis 1:* Greater self-reported substance use will be associated with both lower physical and mental health functioning during COVID-19 for Veterans.

*Hypothesis 2:* Greater levels of negative COVID-19 impacts on quality of life (e.g., finances and meeting basic needs) and COVID-19-related loneliness will each have a distinctive, significant negative relationship with physical and mental health functioning, even when modeled alongside problematic substance use, resulting in a significant increase in variance explained in functioning.

*Hypothesis 3:* Loneliness was hypothesized to moderate the relationship between the negative impact of COVID-19 and both physical and mental health functioning, such that individuals reporting higher levels of loneliness and higher levels of negative COVID-19 impacts on quality of life would report lower mental and physical health functioning, compared to those with higher levels of negative COVID-19 impacts on quality of life but lower levels of loneliness.

## Materials and Methods

### Participants and Procedure

Procedures and methods for collecting this cross-sectional data were completed in accordance with a protocol approved by the Institutional Review Board at the VA Bedford Healthcare System. The survey was administered using the Qualtrics federal platform between November 24, 2020 and February 2, 2021, and United States veterans were identified *via* a Qualtrics panel. Study staff coordinated with a project coordinator from Qualtrics, who used their panel aggregator system, an internal Qualtrics system where over 20 Web-based panel providers have been identified, screened, and utilized by Qualtrics recruiters to supply diverse, quality respondents depending on survey inclusion/exclusion criteria. Research has supported the Qualtrics panel recruitment methodology as an effective recruitment strategy, unlike non-panel recruited anonymous online samples, with evidence supporting that these panels can produce higher quality samples due to internal quality control and prescreened respondents (Ibarra et al., 2018). This data collection method is thus becoming increasingly popular, with evidence showing data quality from the Qualtrics panel is on par with data from conventional data collection methods (e.g., Walter et al., 2019). Potential panel participants were provided with a link to a description of the study and an eligibility survey on the Qualtrics platform. Informed consent was provided and obtained prior to accessing the survey. The survey was estimated to take approximately 30 minutes to complete.

The eligibility criteria for the survey and included as pre-survey screening items were (1) reporting a minimum age of 18 years old, (2) problematic drug and alcohol use indicated by Cut Down, Annoyed, Guilty, and Eye Opener (CAGE)-AID minimum score of 1, and (3) being a veteran of the United States Armed Forces. Veteran status was further assessed by asking participants to report on employment/veteran status overall (such that participants were unaware of the veteran eligibility criteria). If they reported being a veteran, respondents were required to report their DD214 (the Certificate of Release or Discharge form) date and the years they served. Participants were excluded when (1) they had previously completed the survey, (2) did not meet the minimum CAGE-AID score of one, (3) did not meet our verification check for United States Veterans (e.g., were active duty military), or (4) were identified as non-attentive or potential survey spammers after quality-review (see below for criteria). To allow for a wide surveying of veterans with possible substance use concerns, responders were not excluded for (1) not having a formal substance use disorder (SUD) diagnosis, or (2) use of prescribed medications, but did have to report problematic use of drugs or alcohol to qualify for the survey.

Given the high possibility that potential recruited responders might be motivated to complete the survey even without being a veteran due to monetary gains, we instituted a series of survey items and validation checks similar to those utilized by Pedersen et al. (2017, 2021) when recruiting veteran community samples. To verify veteran status, an initial screening item asked respondents for their current military status (i.e., active duty, not and have never been a member of the military, a veteran, or other). If respondents answered they were a veteran, they answered a series of additional items to verify veteran status, including when they had served and in which era (e.g., Vietnam Era, August 1964 to April 1975), and branch of service (e.g., Army). They were then asked if they had received a DD214 and, if yes, were routed to indicate their date of discharge. This date needed to reasonably match their reported service era and self-reported age. If answers were inconsistent, these respondents were removed from the survey.

Approximately 58,500 surveys were sent out to panel participants, who were not aware of the contents of the survey prior to selecting it in their dashboard. A total of 6,500 (11.1%) potential participants entered the survey and were provided with the informational sheet and consent document for the survey. Of these, 381 (5.9%) did not consent to take the pre-survey screening items. Of those who consented to participate in the pre-survey screening, 5,366 (87.7%) were automatically screened out for not reporting being a United States veteran (*n* = 3,200) and for not reporting substance abuse issues on the CAGE-AID screener (*n* = 2,166). Following these automatic eligibility checks (*n* = 753), a total of 436 participants completed the survey (completion rate of 58%). Based on proposed guidance for online recruitment quality review by Heffner et al. (2021), potential participants were removed if three of the following four previously identified quality-review criteria were met: (1) self-reported veteran status was deemed impossible when reviewed in conjunction with age, reported military service era, and/or DD214 date, (2) attention check items showed participants reporting gibberish responses to open-ended questions, (3) total time spent on the survey was under 1/3 of median completion time (< 10 min), and (4) response patterns flagged by the Qualtrics system as potential bots using their quality check software (i.e., IP-based “Prevent Ballot Box Stuffing” option, attempts to take the survey multiple times) were reviewed and found to be suspicious (e.g., answering all survey items as the last Likert scale option). A total of 27 (6.2%) potential participants were removed because they did not meet these criteria.

### Study Measures

#### Demographic Characteristics

The present study evaluated several demographic characteristics, including age, gender, sexual orientation, race, ethnicity, income, armed service branch, VHA connection status, and relationship status.

#### Addiction Measures

Substance use concerns and behaviors were assessed in the screening survey and within the main survey. Problematic use of alcohol and other drugs was assessed using the CAGE Adapted to Include Drugs (CAGE-AID; Brown and Rounds, 1995), a validated four-item measure. Per the CAGE-AID protocol, the questionnaire was only given to participants who reported current alcohol or drug use. A minimum score of 1 on the CAGE-AID was used as a screener to participate in the survey, as recommended by the CAGE-AID Consensus Panel of the Substance Abuse and Mental Health Services Administration (SAMHSA) to cast a wider net and identify more patients with substance abuse or addiction issues (Sullivan and Fleming, 2008). Previous research indicates that a CAGE-AID score of one or more indicates a positive screen with a sensitivity of 0.79 and specificity of 0.77, and that scores can accurately classify 78% of substance users (Ashman et al., 2004).

A modified version of the Alcohol, Smoking, and Substance Involvement Screening Test (ASSIST; Humeniuk et al., 2010) was used to descriptively measure substance use frequency, urges, and changes in use during the pandemic for alcohol, tobacco, cannabis, cocaine, stimulants, inhalants, sedatives, hallucinogens, and opioids (four items for each substance). Item #1, assessing any lifetime non-medical use of a specific substance, was retained in its original form. As a main purpose of the current study was to describe potential changes in substance use during COVID, Item #2 was modified to read, “Since the beginning of the COVID-19 pandemic, how often have you used [specific substance]?” Additionally, Item #3 was modified to “Since the beginning of COVID-19, how has your desire or urge to use [specific substance] changed?” and Item #4 was added, “How has your frequency of using [specific substance] changed during COVID-19?,” with the response scale ranging from More Use/Craving, No Change, and Less Use/Craving.

#### Medical Diagnoses

Medical diagnoses were obtained *via* self-report. Several general medical diagnoses were provided, and participants self-reported whether they had ever received an official medical diagnosis from a doctor or healthcare professional. As many of these diagnoses can be long-standing, we did not specify a timeframe for when the condition might have been officially diagnosed. These medical diagnoses included chronic pain, diabetes, insomnia, heart disease, apnea, and seizures.

#### Psychiatric Diagnoses

Psychiatric diagnoses were self-reported using the same criteria as medical diagnosis (e.g., had been diagnosed by a doctor or other healthcare professional specifically) by participants and included SUDs, major depressive disorder, post-traumatic stress disorder (PTSD), anxiety disorder, panic disorder, psychogenic non-epileptic seizures (PNES), schizophrenia, and bipolar disorder.

#### Psychosocial Functioning

The Short-Form Health Survey-12 (SF-12; Ware et al., 1996) is a 12-item measure that assesses mental and physical functioning. The SF-12 has been validated for predicting populations’ mental and physical health without targeting specific health outcomes and has high reliability, including with United States veterans (Salyers et al., 2000). Scoring involves using a norm-based algorithm that produces a self-reported mental health composite score (MCS) and physical health composite score (PCS) between 0 and 100 (Jones et al., 2001), with lower scores associated with lower functioning. Test–retest reliability is 0.80 for the PCS and 0.76 for the MCS (Ware et al., 1996).

#### COVID-19 Measures

The present study evaluated the experiences, stressors, and impact of COVID-19 across several areas of interest. A subscale score was created using a modified version of the Pain Management Collaboratory Coronavirus Pandemic (COVID-19) 5-Item Measure (PMC-5), which measured the potential negative impact of COVID-19 on quality of life on a scale of 1 (Improved) to 4 (A Lot Worse). The five areas measured by the PMC-5 included finances, emotional health, ability to meet basic needs, and two additional items on physical health and concentration. For the present study, internal consistency for the PMC-5 was satisfactory (five items: *α* = 0.82).

In addition, three modified questions from the Osteoporotic Fractures in Men (MROS) Study COVID-19 Social Impact Questionnaire (Cawthon et al., 2020) with multiple check-box options were asked to describe COVID-19 experiences, including (1) “Which of the following statements best describes your personal experience with the coronavirus,” (2) “Which of the following have you done in the last month to keep yourself safe from coronavirus?,” and (3) “Which of the following are worries of yours related to COVID-19, or are more difficult for you now because of the pandemic?”

#### Loneliness During COVID-19

The UCLA Loneliness Scale-8 (ULS-8; Hays and DiMatteo, 1987) was used to assess feelings loneliness during the pandemic. The scale instructions were slightly modified to assess pandemic-related loneliness and read, “Indicate how often you have felt the following ways during your COVID-19 experience.” The full eight-item measure was utilized, and sample items include “There is no one I can turn to” and “I lack companionship.” Scores range on a scale from 1 (Never) to 4 (Often), with higher scores indicating greater levels of loneliness. Internal validity for this scale in the current sample was satisfactory (eight items: *α* = 0.83).

### Data Analysis

Means, SDs, and frequencies were calculated for the full sample. Zero-order correlations were first conducted among all the measures of interest to assess for initial relationships among model variables. To research the unique effects of each potential predictor, two hierarchical regression analyses were conducted for mental and physical health functioning. The hypothesized model first explored the relationship between problematic substance (CAGE-AID) use on mental health and physical functioning, respectively. In addition, by adding variables related to SDH factors (PMC5 and ULS-8) to the model, we anticipated greater predictive ability for both physical and mental health functioning, with problematic substance use still accounting for a significant amount of variance in functioning. Hierarchical or sequential regression allows for the analysis of multiple predictors when the order of entry is determined by a theoretical base, while also determining the contribution of each variable after controlling for those entered earlier in the model (Tabachnick and Fidell, 2007). In the first step, demographic control variables were included, showing significant zero-order correlations with the dependent variables (gender, age, and annual income). In the second step of the hierarchical multiple regression analysis to test Hypotheses 1, problematic alcohol and drug use concerns (CAGE-AID scores) were entered to investigate their impact on mental health. In the third step, we evaluated our proposed Hypothesis 2 by adding pandemic-related stressors Hierarchical or sequential regression allows for the analysis of multiple predictors when the order of entry is determined by a theoretical base, while also determining the contribution of each variable after controlling for those entered earlier in the model (Tabachnick and Fidell, 2007). In the first step, demographic control variables were included, showing significant zero-order correlations with the dependent variables (gender, age, and annual income). Hypothesis 1 was tested in the second step of the hierarchical multiple regression analysis, with problematic alcohol and drug use concerns (CAGE-AID scores) entered to investigate their impact on mental health (MCS-12). Hypothesis 2 was explored in the third step, with pandemic-related stressors (PMC-5 and ULS-8) added to the model to assess the unique impact of COVID-19 associated SDH factors on mental health functioning. A second hierarchical regression was conducted with the same variables and stepwise model to predict physical health. We tested the assumptions of regression analysis and for evidence of violations. Multicollinearity among predictor variables was set at zero-order correlations greater than 0.70, and continuous variables scores and errors were inspected for normalcy of distribution. In addition, to assess post-hoc Hypothesis 3, exploring a potential interaction between the ULS-8 and PMC5 on functioning, predictors variables were mean-centered to create a cross-product to regress on both outcome variables of interest in the final step. All tests were two-tailed, with all analyses conducted using SPSS v.26.

## Results

### Survey Respondents

The mean age of the sample (*N* = 409) was 54.85 years (*SD* = 16.44), with 76.5% (313/409) of participants reporting a male gender identity. Participants could identify with multiple racial categories, with 90% (370/409) identifying as white/Caucasian, 5.4% (22/409) as Black/African American, and 1.2% (5/409) identifying as American Indian or Alaska Native, Asian, or Native Hawaiian or Pacific Islander, respectively. Additionally, 8.1% (376/409) of participants identified as Hispanic. A total of 91.4% (374/409) of participants reported identifying as straight/heterosexual, and a majority (66.7%; 273/409) of participants were married or living with a partner. See Table 1 for detailed demographic information.

**Table 1:** Sample demographics (*N* = 409).

Variable	Frequency (%)	Variable	Frequency (%)
*Gender*		*Income*	
Male	313 (76.5%)	Less than $19,999	31 (7.60%)
Female	94 (23%)	$20,000–39,999	70 (17.11%)
Transgender Male	1 (0.2%)	$40,000–59,999	66 (16.14%)
Preferred not to answer	1 (0.2%)	$60,000–79,999	53 (12.96%)
Age	*m* = 54.85(*SD* = 16)	$80,000–99,999	48 (11.74%)
Race^*^		$100,000–149,999	87 (21.30%)
White	370 (90.50%)	$150,000 +	54 (13.20%)
Black/African American	22 (5.40%)	*Relationship status*	
Other	7 (1.70%)	Married	273 (66.70%)
Asian	5 (1.20%)	Divorced	47 (11.50%)
Native Hawaiian/Pacific Islander	5 (1.20%)	Single, never married	39 (9.50%)
American Indian/Alaska Native	5 (1.20%)	In a relationship, not married	24 (5.90%)
*Ethnicity*		Widowed	15 (3.70%)
Not Hispanic/Latino	376 (91.90%)	Separated	11 (2.70%)
Hispanic/Latino	33 (8.10%)	Service Branch^*^	
*Sexual Orientation*		Army	202 (49.40%)
Heterosexual (straight)	374 (91.40%)	Air Force	81 (19.80%)
Bisexual	19 (4.6%)	Navy	65 (15.90%)
Homosexual (gay)	13 (3.2%)	Marines	44 (10.80%)
Prefer not to say	3 (0.70%)	National Guard	29 (7.10%)
Service Era^*^		National Reserve	11 (2.70%)
September 2001 or later	156 (38.1%)	Coast Guard	7 (1.70%)
August 1990–August 2001	96 (23.5%)	Army Reserves	1 (0.20%)
May 1975–July 1990	99 (24.2%)	Connected to VHA Care	268 (65.5%)
Vietnam Era (1964–1975)	150 (36.7%)		
February 1955–July 1964	24 (5.9%)		
Korean War (1950–1955)	3 (0.7%)		

* Participants could choose multiple categories.

### Descriptive Results

Of the 409 participants screened into the study with problematic substance use, a minority reported a formal, medically diagnosed alcohol (27.6%; 113/409) or substance use (20.5%; 84/409) disorder. The most reported substances for those with medically diagnosed SUD other than alcohol were tobacco and cannabis. The most reported psychiatric diagnoses were anxiety disorder (36.4%; 149/409), PTSD (27.4%; 112/409), and major depressive disorder (20.3%; 83/409); the most reported physical condition was chronic pain (31.5%; 129/409: see Table 2).

**Table 2:** Veteran sample substance use, psychiatric, and physical health diagnoses.

Substance use diagnoses^*^	Frequency (%)
Alcohol Use Disorder	113 (27.6%)
Substance Use Disorder	84 (20.5%)
*Tobacco Use Disorder*	*62 (73.8%)*
*Cannabis Use Disorder*	*47 (55.9%)*
*Sedative Use Disorder*	*31 (36.9%)*
*Opioid Use Disorder*	*26 (31.0%)*
*Cocaine Use Disorder*	*22 (26.2%)*
*Amphetamine-type stimulant Use Disorder*	*20 (23.8%)*
*Inhalant Use Disorder*	*5 (6.0%)*
*Hallucinogen Use Disorder*	*2 (2.4%)*
**Psychiatric Diagnoses** ^*^	**Frequency (%)**
Anxiety Disorder	149 (36.4%)
Post-Traumatic Stress Disorder	112 (27.4%)
Major Depressive Disorder	83 (20.3%)
Panic Disorder	48 (11.7%)
Bipolar Disorder	31 (7.6%)
Schizophrenia	9 (2.2%)
Psychogenic non-epileptic seizures (PNES)	7 (1.7%)
**Physical Health Diagnoses** ^*^	**Frequency (%)**
Chronic Pain	129 (31.5%)
Diabetes	73 (17.8%)
Insomnia	70 (17.1%)
Heart Disease	48 (11.7%)
Apnea	47 (11.5%)
Seizures	16 (3.9%)

* Participants could choose multiple categories.

Regarding alcohol and non-prescription substance use during COVID-19, a substantial number of participants reported daily or mostly daily use of substances. Of the participants who reported at least some lifetime use of a particular substance, alcohol (45.8%; 182/397) was the most commonly used substance on a daily or mostly daily basis, followed by tobacco (44.9%; 150/334), sedatives (29.8%; 48/161), and opioids (26.6%; 29/109). Participants with current substance or alcohol use also reported changes in cravings during the COVID-19 pandemic. The majority reported no changes in urges/cravings of substances, apart from alcohol, with 47.6% (189/397) reporting an increased urge to drink. For all other substances, the largest increases in cravings were reported for cannabis (38.4%; 98/255) and tobacco (37.1%; 124/334). Finally, many participants reported increased substance use during COVID-19, with the highest increased use for alcohol (45.3%; 180/397). See Table 3 for specific rates by substance.

**Table 3:** Alcohol and non-prescription substance use, urges, and changes in use due to COVID-19.

	Alcohol	Tobacco	Cannabis	Sedatives	Opioids	Stimulants	Cocaine	Inhalants	Hallucinogen
Lifetime/Any Use	397 (97.1%)	334 (81.7%)	255 (62.3%)	161 (39.4%)	109 (26.7%)	105 (25.7%)	95 (23.2%)	42 (10.3%)	74 (18.1%)
*Using during COVID*									
Not at all	39 (9.8%)	113 (33.8%)	84 (32.9%)	23 (14.3%)	34 (21.2%)	39 (37.1%)	49 (51.6%)	6 (14.3%)	39 (52.7%)
Once or twice	16 (4.0%)	20 (6.0%)	26 (10.2%)	19 (11.8%)	17 (15.6%)	18 (17.1%)	10 (10.5%)	9 (21.4%)	7 (9.5%)
Monthly	29 (7.3%)	16 (4.8%)	25 (9.8%)	16 (9.9%)	13 (11.9%)	11 (10.5%)	9 (9.5%)	5 (11.9%)	6 (8.1%)
Weekly	131 (33.0%)	35(10.5%)	60 (23.5%)	55 (34.2%)	16 (14.7%)	21 (20.0%)	15 (15.8%)	16 (38.1%)	17 (23.0%)
Daily or mostly daily	182 (45.8%)	150 (44.9%)	60 (23.5%)	48 (29.8%)	29 (26.6%)	16 (15.2%)	12 (12.6%)	6 (14.3%)	5 (6.8%)
Changes in urge/craving^*^									
Lower urge/craving	54 (13.6%)	43 (12.9%)	32 (12.5%)	34 (21.2%)	16 (14.7%)	18 (17.1%)	10 (10.5%)	13 (31.0%)	6 (8.1%)
No change in urge/craving	154 (38.8%)	167 (50.0%)	125 (49.0%)	68 (42.2%)	55 (50.5%)	52 (49.5%)	59 (62.1%)	16 (38.1%)	49 (66.2%)
Higher urge/craving	189 (47.6%)	124 (37.1%)	98 (38.4%)	59 (36.6%)	38 (34.9%)	35 (33.3%)	26 (27.4%)	13 (31.0%)	19 (25.7%)
Changes in use^*^									
Decreased use	68 (17.1%)	49 (14.7%)	32 (12.5%)	30 (18.6%)	19 (17.4%)	21 (20.0%)	11 (11.6%)	12 (28.6%)	5 (6.8%)
No change in use	149 (36.4%)	168 (50.3%)	134 (52.5%)	72 (44.7%)	60 (55.0%)	51 (48.6%)	61 (64.2%)	15 (35.7%)	50 (67.6%)
Increased use	180 (45.3%)	117 (35.0%)	89 (34.9%)	59 (36.6%)	30 (27.5%)	33 (31.4%)	23 (24.2%)	15 (35.7%)	19 (25.7%)

* Percentage of changes in urges and use based only on participants reporting lifetime/any use for the specific substance.

Participants reported on their specific COVID-19 experiences, with 10.5% (43/409) reporting being officially diagnosed with COVID-19 and 12.7% (52/409) believing they had COVID-19 but were not officially diagnosed (see Table 4). The most frequently reported stressors related to the pandemic were fear of getting COVID-19 (61.4%; 251/409) or someone close to the participant getting COVID-19 (58.7%; 240/409). In terms of safety behaviors, the most common behaviors were wearing a facemask (88.8%; 363/409), avoiding public spaces and crowds (69.2%; 283/409), and avoiding in-person contact with friends and family (50.1%; 205/409).

**Table 4:** Veteran experiences with COVID-19 health, stressors, and safety behaviors.

COVID-19 infection experiences	Frequency (%)
Received COVID-19 medical diagnosis	43 (10.5%)
Believe they had COVID-19, not officially diagnosed	52 (12.7%)
Average number of days experiencing symptoms	11.3 (*SD* = 23.8)
Hospitalized due to COVID-19	2 (0.5%)
Received COVID-19 vaccine^a^	14 (3.4%)
A family member/close friend was diagnosed	136 (33.3%)
A family member/close friend died	53 (13%)
COVID-19 Stressors	Frequency (%)
Getting COVID-19	251 (61.4%)
Someone close to participant getting COVID-19	240 (58.7%)
Feeling isolated and alone	148 (36.2%)
Worries about finances/income	134 (32.8%)
Not getting necessary medical care	99 (24.2%)
Meeting basic needs (e.g., food, housing)	92 (22.5%)
Having difficulty meeting conditions of probation/parole	16 (3.9%)
No worries or stressors related to COVID-19	6 (1.4%)
COVID-19 Safety Behaviors	Frequency (%)
Wore a facemask	363 (88.8%)
Avoided public spaces/crowds	283 (69.2%)
Avoided in-person contact with friends/family	205 (50.1%)
Cancelled/postponed travel	197 (48.2%)
Had a telehealth visit	136 (33.3%)
Had an in-person healthcare visit	124 (30.3%)
Stockpiled food and/or water	118 (28.9%)
Teleworked	101 (24.7%)
Prayed/meditated/engaged in spiritual practice	96 (23.5%)
Cancelled a healthcare appointment	73 (17.8%)
Cancelled/postponed work or school activities	51 (12.5%)
No safety behaviors used	7 (1.7%)

a Vaccination item added 14/12/2021, available to 75.8% of participants.

### Hierarchical Multiple Regression

#### Mental Health Functioning

Prior to multiple regressions, continuous outcome variables were inspected for normality, and all continuous variables had an approximate normal distribution with no significant outliers. In addition, control variables, predictors, and outcome variables were investigated using zero-order correlations (Table 5), which revealed that being young, having greater problematic substance use, reporting more negative COVID-19 impacts on quality of life, and experiencing greater reported loneliness were correlated with lower scores in both mental and physical health functioning. These statistically significant relationships among variables did not suggest multicollinearity (absolute correlation coefficient greater than 0.70).

**Table 5:** Correlations among continuous predictor variables.

Measure	1	2	3	4	5	6
1. Age	-					
2. CAGE-AID	−0.33^***^	-				
3. PMC-5	−0.31^***^	0.31^***^				
4. ULS-8	−0.28^***^	0.28^***^	−0.49^***^			
5. PCS-12	0.10^*^	−0.14^**^	−0.24^**^	−0.24^***^		
6. MCS-12	0.28^***^	−0.24^***^	−0.33^***^	−0.45^***^	−0.19^**^	--
*Mean*	54.84	2.37	2.46	18.30	43.31	42.48
*Median*	56.00	2.00	2.40	18.00	43.95	43.06
*SD*	16.44	1.12	0.57	5.44	6.79	6.78
Range	19–88	1–4	1–4	8–32	24.56–59.81	19.91–63.72

CAGE-AID, CAGE adapted to include drugs scale; ULS-8 Loneliness, UCLA Loneliness Scale-8; PMC-5, Pandemic Impact: Pain Management Collaboratory (PMC) 5-Item COVID Negative Impact Measure; MCS-12, Short-Form Health Survey’s (SF-12) Mental health component scale; and PCS-12, Short-Form Health Survey’s (SF-12) Physical health component scale.

* *p* < 0.05,

** *p* < 0.01, and

*** *p* < 0.001.

Following the investigation of these correlations, hierarchical multiple regression analyses were conducted. In the first step of the regression, age, gender, and race were entered as covariates and explained a significant amount of the variance in mental health functioning, *F*(3, 403) = 13.83, *p* < 0.001, adjusted *R*^2^ = 0.09. In the second step, results from the CAGE-AID were entered to control for the impact of problematic drug and alcohol use on mental health outcomes, and explained a significant amount of variance, *F*(4, 402) = 13.41, *p* < 0.001 adjusted *R*^2^ = 0.12, with CAGE-AID score explaining a significant additional amount in variance in mental health functioning (*β* = −0.17, *p* = 0.001), such that greater levels of problematic use of substances predicted decreases in mental health. In the third and final step, indicators of COVID-19-related impacts, specifically the PMC-5 (negative COVID-19 impacts on quality of life) and ULS-8 (pandemic-related loneliness), were entered. This addition resulted in a significant change in *R^2^* of 0.12 (*p* < 0.001), with PMC-5 (*β* = −0.12, *p* = 0.03) and ULS-8 scores (*β* = −0.34, *p* < 0.001) as significant predictors of decreased mental health functioning during COVID-19. With the addition of these pandemic stressor variables, CAGE-AID scores no longer predicted mental health functioning (*β* = −0.07, *p* = 0.14). There was no significant interaction between ULS-8 and PMC-5 on mental health functioning (*β* = −0.05, *p* = 0.30). This finding indicates that negative COVID-19 impacts on quality of life (PMC-5) and pandemic-related loneliness (ULS-8) had a unique and negative impact on veterans’ mental health functioning (MCS-12). The entire model accounted for 23% of the variance in the MCS (see Table 6).

**Table 6:** Summary of hierarchical regression analysis predicting mental and physical health functioning (*n* = 409).

Step and Variable	Mental Health Functioning					Physical Health Functioning				
	*B*	*SE*	*β*	*sr* ^2^	Adjusted *R*^2^	*B*	*SE*	*β*	*sr* ^2^	Adjusted *R*^2^
Step 1					0.09^***^					0.04^***^
Age	0.11	0.02	0.27	0.07^***^		0.05	0.02	0.09	0.02^*^	
Gender	1.30	0.81	0.08	0.01		0.16	0.84	0.01	0.00	
Income	0.14	0.10	0.07	0.00		0.36	0.10	0.18	0.03^***^	
Step 2					0.12^***^					0.04^***^
Age	0.09	0.02	0.21	0.02^***^		0.04	0.02	0.09	0.01	
Gender	1.50	0.80	0.09	0.01		0.28	0.84	0.02	0.00	
Income	0.11	0.10	0.05	0.00		0.34	0.10	0.17	0.03^**^	
CAGE-AID	−1.00	0.30	−0.17	0.02^**^		−0.62	0.31	−0.10	0.01*	
Step 3					0.23^***^					0.08^***^
Age	0.05	0.02	0.12	0.01^*^		0.02	0.02	0.04	0.00	
Gender	0.38	0.76	0.02	0.00		−0.37	0.84	−0.02	0.00	
Income	−0.07	0.09	−0.04	0.00		0.23	0.10	0.12	0.01^*^	
CAGE-AID	−0.43	0.29	−0.07	0.00		−0.28	0.32	−0.05	0.00	
ULS-8	−0.42	0.07	−0.34	0.08^***^		−0.18	0.07	−0.14	0.01^*^	
PMC-5	−1.37	0.63	−0.12	0.01^*^		−1.35	0.69	−0.11	0.01^*^	
PMC-5 × ULS-8	−0.97	0.09	−0.05	0.30		0.07	0.10	0.03	0.51	

CAGE-AID, CAGE adapted to include drugs scale; ULS-8: Loneliness, UCLA Loneliness Scale-8; and PMC-5, Pandemic Impact: Pain Management Collaboratory (PMC) 5-Item COVID Negative Impact Measure.

* *p* < 0.05,

** *p* < 0.01, and

*** *p* < 0.001.

#### Physical Health Functioning

To begin testing the replicability of the previously outlined hierarchical multiple regression model on physical health, age, gender, and race were entered as covariates, which explained a significant amount of the variance in physical health functioning, *F*(3, 403) = 6.25, *p* < 0.001, adjusted *R*^2^ = 0.04. In the second step, the CAGE-AID was entered into the model and explained a significant amount of variance, *F*(4, 402) = 5.70, *p* < 0.001, adjusted *R*^2^ = 0.04 in physical health functioning (*β* = −0.10, *p* = 0.04), such that greater problematic use of substances predicted decreases in physical health. In the third step, indicators of COVID-19 related-impacts were entered. This addition resulted in a significant change in *R^2^* = 0.04, *p* < 0.001, and although CAGE-AID scores no longer predicted physical health (*β* = −0.05, *p* = 0.38), negative COVID-19 impacts on quality of life (PMC-5; *β* = −0.11, *p* = 0.03), and pandemic-related loneliness scores (ULS-8; *β* = −0.14, *p* = 0.02) emerged as significant predictors of decreased physical health functioning during COVID-19. There was no significant interaction between ULS-8 and PMC-5 on physical health functioning (*β* = 0.03, *p* = 0.51). This finding indicates that stressors from the pandemic, both loneliness and perceived negative impact of COVID-19 on quality of life, had a unique and negative impact on veterans’ physical health functioning during COVID-19. The entire model accounted for 9% of the variance in the physical functioning composite score.

## Discussion

The primary aim of this study was to describe the experience of the COVID-19 pandemic on United States veterans reporting problematic substance use and model the pandemic’s impact on functioning. Overall, results showed that physical and mental health functioning during COVID-19 had been strongly and negatively impacted by pandemic-related decreases in important life domains and quarantine-related loneliness for veterans reporting substance use issues. As pandemic infection and death rates continue to wane and surge, such findings are important in understanding the unique issues that might arise for veterans in our community dealing with substance use concerns.

### Pandemic Experiences, Behaviors, and Stressors for United States Veterans

To investigate our primary descriptive aim, United States veteran participants reported on a broad range of pandemic-related health experiences and safety behaviors during the COVID-19 pandemic. Though only 23% had a confirmed or assumed COVID-19 diagnosis at the time of this survey, a third had experienced a family member or close friend becoming infected. Additionally, of those without reported coronavirus, nearly all participants sampled reported stress related to themselves or someone close to them becoming infected. Given these concerns, it is unsurprising that there was a high rate of reported safety behaviors, including mask-wearing and social distancing—although not all participants reported these behaviors. This finding suggests that similar to civilian populations (Knowles and Olatunji, 2021), there has been a wide range of accepted and applied COVID-19 infection-control activities within the United States veteran population. In addition, only about a third of the participants had attended at least one healthcare appointment (either telehealth or in-person), and 17.8% had canceled a healthcare appointment due to COVID-19. As 35% of our sample was not receiving healthcare at the VA and given the low rates of help-seeking yet high rates of comorbid mental and physical health concerns in the veteran population, this finding was particularly concerning. This disruption of healthcare services during COVID-19 is ongoing and suggests that the VHA should make strong attempts to enroll unconnected community veterans across the United States or reconnect with veterans who may have lapsed in their VHA healthcare utilization during the pandemic.

Our results also emphasized that many United States veterans reporting problematic substance use over the past year had increased their alcohol and non-prescription drug use during COVID-19 and attributed this increase specifically to the pandemic. This finding was most pronounced in drinking, where most participants reported an increase in drinking during the pandemic. Although all participants self-reported at least some problematic drug or alcohol use for study inclusion, only about a third of participants of our sample reported a formal substance use diagnosis. This discrepancy coincides with research showing problematic use of substances is perhaps more ubiquitous than during non-pandemic times (Satre et al., 2020), possibly as a means of coping with pandemic-related stressors (Wardell et al., 2020). However, other research suggests the COVID-19 related increases in substance use vary for different veteran populations, such as those with pre-COVID mental health concerns being associated with increased drinking (Davis et al., 2021; Pedersen et al., 2021). From a clinical perspective, this increase in substance use may be explained by additional underlying psychological mechanisms, such as cognitive process (e.g., Desire Thinking; Mansueto et al., 2019), psychiatric symptom management (Cosci et al., 2019), personality vulnerabilities such as impulsive sensation-seeking or behavioral disinhibition (Sher et al., 2000; Oh et al., 2021), and dysfunctional coping strategies (see Cavicchioli et al., 2018). These should be assessed in the future, to both investigate what factors may maintain increased COVID-19 substance use and as potential therapeutic targets for addictive behaviors interventions. Given our finding of increased substance use for many veterans, providers should be even more committed to screening patients, especially those with diagnosed mental health disorders, for potential SUDs during our continued COVID-19 pandemic management.

### The Relationship Among Substance Issues, Pandemic Experiences, and Loneliness

To explore the physical and mental health functioning of these United States veterans during the pandemic, we investigated several potentially relevant variables hypothesized to be significantly associated with functioning. Consistent with our first hypotheses, higher subjective drug and alcohol concerns were related to lower mental and physical health functioning, similar to other research on substance misuse and functioning (e.g., Sheckter et al., 2020). Our second hypothesis, that negative COVID-19 impacts on quality of life (e.g., finances and meeting basic needs), greater self-reporting of problematic substance use, and COVID-19-related loneliness will have each a distinctive, significant negative physical and mental health functioning, was only partially supported by our data. Findings revealed COVID-19 related pandemic impacts and loneliness were negatively associated with both mental and physical functioning; indicating that distress due to the COVID-19 pandemic has strongly impacted both areas of functioning. This finding aligns with ongoing research into pandemic stressors and their impact on quality of life (Yu et al., 2021). Researchers are likely underestimating the negative impact of COVID-19 across multiple life domains and social isolation on United States veterans with SUD concerns in terms of functioning, treatment, and relapse rates (Linas et al., 2021). Our findings show that there have been serious, negative impacts reported by United States veterans due to COVID-19 on quality of life, social relationships, and substance use. Continued research during and beyond the pandemic should account for these stressors and model their long-term impact on SUD behaviors and coping.

Our final regression model assessing our second hypothesis highlighted that, when considered in conjunction with pandemic-related quality of life and COVID-19 related loneliness, problematic substance use behaviors alone did not significantly predict either mental or physical health functioning. Our *post hoc* interaction analysis, exploring the possibility that the negative impact of COVID-19 on quality of life and its relation to functioning might be moderated by reported levels of loneliness, was not supported. Instead, the final model highlighted the strong, main effect of negative COVID-19 impacts on quality of life and self-reported loneliness on decreased functioning for these veterans reporting problematic substance use concerns. The impact of COVID-19 related quality of life factors, such as financial well-being and being able to meet basic needs, emphasized the importance of attending to changes in SDH factors and environmental contexts when considering the physical and psychological functioning of veterans with substance use concerns. The strong association between loneliness and both physical and mental health functioning was also particularly important. This aligns with pre-pandemic evidence showing social support being associated with decreased all-cause mortality, cardiovascular disease, depression, and anxiety (Leigh-Hunt et al., 2017), and pandemic-specific research showing loneliness as a strong predictor of worse functioning, affective illness, and non-accidental self-injury (Greig et al., 2022). As loneliness can lead to worse outcomes, effective interventions that are also consistent with COVID-19 infection control and social distancing measures, such as those suggested in the systematic review by Williams et al. (2021), may be of particular importance in veteran treatment.

Given these findings, it is recommended that ongoing assessments of U.S. veterans’ mental and physical health functioning should evaluate not only addictive behaviors but also pandemic-related experiences and SDH. Particular emphasis should be placed upon assessing veterans’ basic needs, financial insecurity, and loneliness, as these may be particularly predictive of physical and mental health functioning. These data align with decades of research into the importance of SDH impacting individuals with substance use concerns (Rhodes, 2002; Collins et al., 2019), highlighting that these factors may be even stronger determinants of functioning in the context of a global pandemic (Russell et al., 2021).

### Limitations

A major limitation with this anonymous survey was having only self-reported data. First, all diagnoses were collected *via* participant self-report, with no diagnostic evaluation or medical record review conducted to confirm veteran’s reported psychiatric, medical, and SUD diagnoses. Though a clinically validated screening instrument (CAGE-AID) was used to assess for problematic drug and alcohol use concerns, a more rigorous evaluation of substance use diagnostic criteria would have strengthened the study. Second, as changes in the frequency of use and cravings related to alcohol and drugs were also self-reported, responses might be subject to recall, response, and social desirability biases. Yet, most surveys are limited to recall limitations, and given the online and anonymous nature of the survey, biases in response and social desirability are likely low. In addition, generalizability of the current sample may be limited due to a sample that was less ethnically and racially diverse than the national United States veteran sample (i.e., sample is 90% White/Caucasian, whereas nationally, White/Caucasian veterans are 80% of the veteran population). Using a panel-recruited, web-based survey methodology may have been susceptible to fraudulent and biased responses. The research team implemented standardized quality control reviews, veteran status verification procedures, and data inclusion screening procedures to minimize this concern, including analysis of click-through behavior and scrubbing methods for web-based panel quality, resulting in higher confidence related to survey response quality. A final and important limitation of our study is that it was cross-sectional. We are thus unable to assess directionality or causality of the relationship between variables, limiting our ability to predict changes in substance use or functioning outside of participant self-report of such changes. Although logistic regressions to investigate COVID-19 related factors impacting changes in substance use may have added to this understanding, this analysis is beyond the scope of the current paper and will be examined in future analyses.

## Conclusion

The level of anxiety and stress that the COVID-19 global pandemic has inflicted on the world is staggering. United States veterans struggling with alcohol and substance addiction have reported significant increases in substance use and feelings of pandemic-related anxiety. In addition to substance-use related concerns, pandemic-related stressors and loneliness have further diminished these veterans’ physical and mental health. As the spread of vaccines curtails the potential impact of COVID-19, increased research on the mental health impact of the months-long lockdowns, variant impacts, and possibly years-long social distancing protocols have had on individuals suffering from substance use issues will need to be investigated. Future research using a community sample of United States veterans would benefit from both continued, longitudinal data collection and additional assessments of drug and alcohol use (e.g., the AUDIT or DAST). Additionally, future work should further investigate the underlying mechanisms of substance use changes, as well as moderating factors related to managing allostatic load and the impact of SDH during the pandemic, such as psychological flexibility. This would allow for the assessment of alcohol and drug use disorders diagnostically, while also modeling the impact of resiliency factors on health and functioning trajectories in this population. A better understanding of how veterans with self-reported problematic substance use have been both struggling and successfully navigating their unique COVID-19 societal conditions may provide further information on areas of future interventions.

## Data Availability Statement

The raw data supporting the conclusions of this article will be made available by the authors, without undue reservation.

## Ethics Statement

The studies involving human participants were reviewed and approved by VA Bedford Healthcare System Institutional Review Board. The patients/participants provided their informed consent to participate in this study.

## Author Contributions

ER, EC, BD, SS, JH, and MK conceived the study, provided conceptual guidance, and survey distribution procedures and data collection and contributed to writing the manuscript. ER organized, cleaned, and initially analyzed the data. ER, MK, and SS interpreted the data. All authors contributed to the article and approved the submitted version.

## Funding

This work was supported by funds from the VISN 1 New England Mental Illness Research, Education, and Clinical Center (MIRECC) for COVID-19 veteran research (PI: ER). The findings and interpretations of the data expressed in the article are the sole responsibility of the authors and do not necessarily represent the views of the Department of Veterans Affairs.

## Conflict of Interest

The authors declare that the research was conducted in the absence of any commercial or financial relationships that could be construed as a potential conflict of interest.

## Publisher’s Note

All claims expressed in this article are solely those of the authors and do not necessarily represent those of their affiliated organizations, or those of the publisher, the editors and the reviewers. Any product that may be evaluated in this article, or claim that may be made by its manufacturer, is not guaranteed or endorsed by the publisher.
